# Alterations in the Cell Cycle in the Cerebellum of Hyperbilirubinemic Gunn Rat: A Possible Link with Apoptosis?

**DOI:** 10.1371/journal.pone.0079073

**Published:** 2013-11-01

**Authors:** María Celeste Robert, Giulia Furlan, Natalia Rosso, Sabrina Eliana Gambaro, Faina Apitsionak, Eleonora Vianello, Claudio Tiribelli, Silvia Gazzin

**Affiliations:** 1 Fondazione Italiana Fegato (Italian Liver Foundation, Centro Studi Fegato), Trieste, Italy; 2 Department of Medical Sciences, University of Trieste, Trieste, Italy; University of S. Florida College of Medicine, United States of America

## Abstract

Severe hyperbilirubinemia causes neurological damage both in humans and rodents. The hyperbilirubinemic Gunn rat shows a marked cerebellar hypoplasia. More recently bilirubin ability to arrest the cell cycle progression in vascular smooth muscle, tumour cells, and, more importantly, cultured neurons has been demonstrated. However, the involvement of cell cycle perturbation in the development of cerebellar hypoplasia was never investigated before. We explored the effect of sustained spontaneous hyperbilirubinemia on cell cycle progression and apoptosis in whole cerebella dissected from 9 day old Gunn rat by Real Time PCR, Western blot and FACS analysis. The cerebellum of the hyperbilirubinemic Gunn rats exhibits an increased cell cycle arrest in the late G0/G1 phase (p < 0.001), characterized by a decrease in the protein expression of cyclin D1 (15%, p < 0.05), cyclin A/A1 (20 and 30%, p < 0.05 and 0.01, respectively) and cyclin dependent kinases2 (25%, p < 0.001). This was associated with a marked increase in the 18 kDa fragment of cyclin E (67%, p < 0.001) which amplifies the apoptotic pathway. In line with this was the increase of the cleaved form of Poly (ADP-ribose) polymerase (54%, p < 0.01) and active Caspase3 (two fold, p < 0.01). These data indicate that the characteristic cerebellar alteration in this developing brain structure of the hyperbilirubinemic Gunn rat may be partly due to cell cycle perturbation and apoptosis related to the high bilirubin concentration in cerebellar tissue mainly affecting granular cells. These two phenomena might be intimately connected.

## Introduction

Crigler-Najjar Syndrome type I (CNI) is a rare deficiency of bilirubin UDP-glucuronosyl-transferase 1A1 (UGT1A1) activity caused by mutations of the *UGT1A1* gene[1,2]. The inability to effectively conjugate unconjugated bilirubin (UCB), results in massive deposition of the pigment in tissues, notably causing neuronal loss in selected structures of the central nervous system early in infancy [3–5].

The homozygous (jj) hyperbilirubinemic Gunn rat [6], manifest an inherited absence of the UDP-glucuronosyl-transferase (Ugt1a1) enzyme activity, due to a spontaneous point mutation in the Ugt1a1 gene, leading to a truncated-inactive protein. With similar (to humans) inability to effectively conjugate and excrete bilirubin and central neurological damage, it has been used as an animal model of CNI and neonatal jaundice [7–9]. In jj Gunn rats, a statistically significant arrest in cerebellar growth develops by postnatal day 9 [10], with prominent loss and degeneration of Purkinje cells and granule neurons, impairment of myelinisation, arborisation and synaptogenesis, and reduction in the molecular and external granular cell layer thickness [11–17]. Similar findings of bilirubin toxicity to cerebellum have been documented also in humans [3,18,19].

UCB is believed to induce damage by altering membrane permeability and transport [20], inducing mitochondrial energy failure [21], and increasing intracellular calcium concentration [22]. These effects are believed to trigger excitotoxicity, redox state imbalance, inflammation and activate apoptosis/necrosis of cells [21,23–26]. Similarly, hyperbilirubinemia has been associated with reduced cholesterol concentrations [27–29]. The brain contein about the 25% of the total body cholesterol, mostly “entrapped” in the myelin (70-80%), and cellular membranes (50-90%) [30]. Cholesterol biosynthesis is needed for myelin production by oligodendrocytes, and recently oligodendrocytes have emerged as the brain cellular population most sensitive to bilirubin toxicity. The altered myelination reported both in the Gunn rat and humans [15,17,18] are in line with the alteration in cholesterol metabolism .

Recently, the high concentration of plasma bilirubin of jj Gunn rat hasbeen associated with the suppression of vascular neointima hyperplasia after balloon injury [31–33] and inhibition of tumour growth [34,35]. *In vitro* treatment of primary cultures and tumoral cell lines with UCB (or its precursor, biliverdin) caused cell cycle arrest in the G1 phase, due to a decrease of Cyclin D, E, A as well as Cdk2 protein [31–36]. Most importantly, perturbation of the cell cycle by bilirubin exposure has been documented in neuronal cells *in vitro* [37].

The macroscopic effect of bilirubin toxicity (hypoplasia) in the brain of the Gunn rat occurs in the cerebellum [10], a CNS structure rapidly developing early after. At this time, proliferation of granular precursor cells localized in the external granular layer, is the dominant event in the cerebellar growth [38,39], making this structure an ideal target for bilirubin toxicity. Functional and morphological findings of bilirubin toxicity to cerebellum have also been documented in humans [3,18,19,40], suggesting the validity of the Gunn rat model in newborn jaundice.

The current study explores *in vivo* a possible links between hyperbilirubinemia, cell cycle perturbation and apoptosis, in relation to the cerebellar hypoplasia of jj Gunn rat pups, as model for the human pathology. Cerebellar tissue of jaundiced (jj) pups and their non-jaundiced (JJ) littermates were compared at post-natal day 9, just at the onset of cerebellar growth arrest in the jj rats. 

## Materials & Methods

### Animals

Animals (of both sexes) were obtained from the animal facility of the Department of Life Sciences of Trieste University. Animal care and procedures were conducted according to the Italian Law (decree 116-92) and to The European Communities Council Directive 2010/63/EU. The project was approved by the Local Ethical Committee of the Trieste University (protocol no: 387 –II/9). Because animals spontaneously develop the pathology, and no treatments have been applied, additional ethical approval was not required. All efforts were made to minimize the number of animals and their suffering. Hyperbilirubinemic Gunn rats (jj) and normobilirubinemic wild-type (JJ) were sacrificed 9 days after birth (P9) by decapitation under (isofluorane) anaesthesia. 

As previously shown by us [10] and others [13], at P9 the cerebellar hypoplasia in Gunn pups is just starting to be appreciable. Only thereafter the slope of growth curve and the cerebellar weight diverge from those of normobilirubinemic littermates indicating that cell death is the major phenomena occurring after P9 to account for cerebellar hypoplasia. For this reason, this post natal stage (P9) was considered the more suitable stage to study the presence both cell cycle perturbation and cell death. The cerebellum was immediately removed and cleaned of meninges and 4th ventricle choroid plexus. In addition cell cycle was evaluated also on the cerebral cortex (a post mitotic CNS region after the birth), dissected from normo-bilirubinemic (JJ) and hyperbilirubinemic (jj) P9 Gunn rats (4 animals each genotype) after cleaning of meninges and lateral ventricle choroid plexuses.

### Bilirubin quantification in plasma and cerebella

Total bilirubin was quantified in plasma by the diazo-reaction method [41] and unconjugated bilirubin (UCB) in whole cerebella by the method developed by Zelenka [42]. Data are expressed as mean ± SD of 6 independent biological repetitions. 

### RNA isolation and real-time PCR analysis

Each hemi-cerebellum (9 animals each genotype. Each animal was individually analysed) was collected in TriReagent (Sigma-Aldrich, St Louis, MO, USA), and total RNA isolated following the manufacturer’s instructions. Retro-transcription of total RNA (1 μg) was performed with an iScript^TM^ cDNA Synthesis Kit (Bio-Rad Laboratories, Hercules, CA, USA) according to the manufacturer’s instructions.

The primers for the targeted genes *Cyclin D1, E1, A, A1 and Cdk2*, selected because reported as modulated by bilirubin [31,32] and the two house-keeping genes (hypoxanthine-guanine phosphoribosyltransferase: *Hprt1* and glyceraldehyde 3-phosphate dehydrogenase: *Gapdh*) were designed using Beacon Designer 8.10 software (PREMIER Biosoft International, Palo Alto, CA, USA) (Table 1). The quantitative analysis of gene expression was performed by real-time PCR on 25 ng of cDNA. The gene-specific sense and anti-sense primers (usually 250 nM, 300 nM for *Cyclin A1* primers) were added with iQ SYBER Green Supermix in an i-Cycler iQ thermocycler (Bio-Rad Laboratories, Hercules, CA, USA), the reaction was performed as previously described [43]. The relative quantification was made using the iCycler iQ software, version 3.1 (Bio-Rad Laboratories, Hercules, CA, USA) by the ΔΔCt method [44,45], taking into account the efficiencies of the individual genes and normalizing the results to the two housekeeping genes. The levels of mRNA were expressed relative to a reference sample.

**Table 1:** Sequence of PCR primers for real-time PCR and efficiency.

**Gene**	**Accession Number**	**Forward**	**Reverse**	**Eff. (%)**
***Gapdh***	NM_017008.3	CTCTCTGCTCCTCCCTGTTC	CACCGACCTTCACCATCTTG	100.0
***Hprt1***	NM_012583.2	AGACTGAAGAGCTACTGTAATGAC	GGCTGTACTGCTTGACCAAG	102.5
***Cyclin D1***	NM_171992.4	AGCTGAGGCGTCCCAACC	CAACCAGAATACACAAAGCCAACC	101.6
***Cyclin E1***	NM_001100821.1	CAGTCAGCCTTGGGATGATGATTC	TCCTCCAGACCTCTTCTCTATTGC	98.6
***Cdk2***	NM_199501.1	CGGCAGGATTTTAGCAAGGTTGTG	CAGGTGAAGAGGGCTTTGGGAAG	98.0
***Cyclin A1***	NM_001011949.1	TATCTTCCTTCCTTGGTAG	TAATGAATAGCCCGTAAATG	106.6
***Cyclin A2***	NM_053702.3	TAGATGCTGACCCATACC	TCCTGTGACTGTGTAGAG	94.9

### Protein isolation and quantification by Western blot analysis

The other hemi-cerebellum (9 animals each genotype) was used for protein analysis as previously described [43]. Total protein content was determined by the bicinchoninic acid kit (Procedure # TPRO 562, Sigma, St Louis, MO, USA). Proteins (25 µg) were separated by SDS-PAGE on 10 or 12% polyacrylamide gels and transferred at 100 V for 1 hr onto PVDF membrane (0.2 µm; Whatman Schleicher & Schuell, Dassel, Germany). PVDF membranes were saturated for 2hr in blocking solution (4% non fat milk or bovine serum albumin in T-TBS) and incubated overnight at 4°C with the appropriate antibody (Table 2). Secondary anti-rabbit or anti-mouse HRP conjugated antibodies (P0448, 0.04 µg/ml, and P0260, 0.26µg/ml, Dako, Glostrup, Denmark) were used. Signal was detected by chemiluminescence (Millipore, Billeria, MA, USA) on X-ray films (BioMax Light, Kodak Rochester, NY, USA), and protein expression obtained as previously described [46]. Differentially, the p18-Cyclin E, Parp-1 (116 kDa), cleaved Parp1 (cParp-1, 89 kDa) and active Caspase-3 (cCasp3, 17 kDa) protein expression (Table 2), were obtained only by normalization with the actin signal on 50 µg of protein. In both cases, actin and targeted protein signals were developed on the same PVDF membrane. The same procedure was applied to the cerebral cortex samples.

**Table 2:** Antibodies used for Western blotting.

	**Blocking Solution**	**Concentration (µg/mL)**	**Antibody**	**Manufacturer**
***Cyclin D1***	4% BSA	135.0	[DCS-6] ab10540	Abcam, Cambridge, UK
***Cyclin E***	4% BSA	2.0	[E-4] SC-25303	Santa Cruz Biotechnology, Santa Cruz, CA, USA
***Cyclin E***	4% non fat milk	2.0	[M-20] SC-481	Santa Cruz Biotechnology, Santa Cruz, CA, USA
***Cyclin A***	4% BSA	0.5	[H-432] SC-751	Santa Cruz Biotechnology, Santa Cruz, CA, USA
***Cdk2***	4% non fat milk	0.03	[M2] SC-163	Santa Cruz Biotechnology, Santa Cruz, CA, USA
***Actin***	4% non fat milk	0.1	A2066	Sigma, St. Louis, MO, USA
***p18-Cyclin E***	4% non fat milk	2.0μg/mL	[M-20] SC-481	Santa Cruz Biotechnology, Santa Cruz, CA, USA
***Parp-1***	4% non fat milk	0.034μg/mL	#9542	Cell Signalling Technology, Danvers, MA, USA
***cCasp-3***	4% non fat milk	0.175 µg/mL	#9662	Cell Signalling Technology, Danvers, MA, USA

### Tissue dispersion and cell cycle analysis

Cell dispersal and fixation was performed on freshly dissected cerebellum as previously described with minor modifications [47,48] (additional 7 JJ and 9 jj Gunn pups). Briefly, small pieces of the tissue were mechanically dispersed by 100 µm and 40 µm nylon cell strainers (BD Falcon # 352560 and # 352540, BD Bioscience, Bedford, USA). After centrifugation (310 g; 4°C; 6 min), the cells pellet were fixed by adding 5 ml of cold (-20°C) 80% ethanol drop-wise under constant gentle vortexing. Samples were incubated for 30 min on ice and stored at -20°C until stained. Thus, cells were stabilized at RT for ^≈^ 10 min. After two sequential centrifugations (310g; RT; 6 min) the sediments were resuspended in 1ml of staining solution in PBS containing 0.1% v/v Triton X-100, 20 µg/ml propidium iodide (PI), and 0.2mg/ml DNAse free RNaseA. Samples were incubated in dark for 30 min at RT and subjected to FACS analysis (cytometer BD FACSCalibur^TM^; and CellQuest software, BD Biosciences, San Jose´, CA). Data were collected for 10,000 events per sample.

### Primary cultures generation

To identify the cell type primarily affected by bilirubin (neuronal vs. glial), primary cultures of granular neurons and astrocytes from freshly dissected Wistar rat cerebella (HanTM - RccHanTM:WIST) were used (Ethical Committee of the Trieste University, protocol no: 387 –II/9). Animals were sacrificed by decapitation under isofluorane anaesthesia. The cerebellum was immediately removed and cleaned of meninges and 4th ventricle choroid plexus. 

Cerebellar Astrocytes Primary Cultures were obtained pooling the cerebella dissected from 6 P2 Wistar pups for each biological repetition (5 biological repetitions), as described previously by Booher [49]. Briefly, the dissected Cll were collected in RT Dulbecco’s Modified Eagle Medium, low glucose (DMEM; Invitrogen, Life Technologies Corporation, Grand Island, NY) supplemented with 10 % heat-inactived foetal bovine serum (FBS; Sigma-Aldrich, St. Louis, MO, USA) and 50 µg/mL gentamycin (Sigma-Aldrich, St. Louis, MO, USA) (astrocytes culture medium). After cutting into small pieces, cells were mechanically dissociated by a 70 µm nylon cell strainer (#352350, BD Biosciences, Corning Inc., Corning, NY, USA) and 20 times pipetting through a 5 mL pipette. The resulting cell suspension was seeded at 1.2 x 10^5^ cells/mL in 6-well plates, and incubated in astrocyte culture medium at 37°C, 5 % CO2, 90 % humidity. Experiments were performed at a confluence of about 90 %. 

Cerebellar Granular Cell Primary Cultures [50], were obtained dissecting the cerebella from 6 P8 Wistar rats a biological repetition (5 biological repetitions). Tissues were collected at RT in BME (Invitrogen, Life Technologies Corporation, Grand Island, NY) medium with 100 µg/mL gentamycin (Sigma-Aldrich, St. Louis, MO, USA), After trituration, the tissue was transferred into 10 mL of solution 1 (0.03 % MgSO4 and 3 mg/mL BSA in Krebs buffer: 124 mM NaCl, 5.37 mM KCl, 1.01 mM NaH2PO4.H2O, 2.7 µM Phenol Red, 25 mM Hepes, 14.5 mM D-glucose, pH 7.40) and centrifuged at 200 g for 1 min. The pellet was resuspended in 10 mL of Solution 2 (0.03 % MgSO4, 3 mg/mL BSA and 0.12 mg/mL trypsin in Krebs buffer) and incubated at 37°C for 15 min with gentle agitation. Trypsin was inhibited by the addition of 10 mL of Solution 3 (0.03 % MgSO4, 3 mg/mL BSA, 0.16 mg/mL trypsin inhibitor, 0.014 mg/mL DNase I in Krebs buffer), and suspension centrifuged at 200 g for 1 min. After decanting of the supernatant, 3 mL of Solution 4 (0.07 % MgSO4, 3 mg/mL BSA, 0.52 mg/mL trypsin inhibitor, 0.045 mg/mL DNase I in Krebs buffer) were added, and cells gently separated by pipetting through a sterile fire-polished glass Pasteur pipette. After 4 % BSA gradient-separation (Gradient Solution: 4% BSA in 0.06 % MgSO4, 0.001 % CaCl2, 3 mg/mL BSA in Krebs buffer), 180 g for 5 min, the cell pellet was resuspended in 5 mL of Neuron Medium (BME, 10 % heat-inactivated FBS, 2 mM L-glutamine, 25 mM KCl, 10 mM Hepes, 44 mM NaHCO3, 100 µg/mL gentamycin, pH 7.40). Cells were seeded in 5 µg /mL poly-L-lysine (molecular weight 30,000-70,000) pre-coated 6-well plate at the density of 1.5 x106 cells/well in Neuron Medium, and incubated at 37°C, 5 % CO2, 90 % humidity. After 24 h the medium was renewed with the addition of cytosine-β-D-arabino-furanoside (Ara-C, 10 µM), to prevent the growth of non-neuronal cells, and D-glucose (5.6 mM). Where not differently indicated, reagent were from Sigma-Aldrich, St. Louis, MO, USA.

### Exposure of primary cell to bilirubin

Both cell types were exposed to 126 nM of free bilirubin (Bf). This value of Bf was selected based on previous data from our group [51–54] and, most importantly, because mimics the Bf found in P9 jj Gunn rats [55–57]. 25 µM purified UCB [58], dissolved in DMSO (vehicle) (Sigma-Aldrich, St. Louis, MO, USA) was diluted in culturing medium, containing 10% FBS. Bf concentration was assessed by peroxidase assay [59,60], and cells exposed for two hours. Control cells were exposed to the same DMSO amounts (0.5% final volume) needed to produce the desired Bf.

### Evaluation of bilirubin effects on cell cycle and apoptosis

FACS analysis of cell cycle on primary cells harvested with trypsin, was performed as described above for the *in vivo* analysis. Apoptosis was evaluated by cCasp3 detection by Western blot (see *in vivo* section).

### Statistical analysis

Significant differences between genotypes were determined by unpaired t-test using InStat 3.06 (GraphPad Software, San Diego, CA, USA). Differences were considered statistically significant at a p value lower than 0.05. All results were expressed as mean ± S.D.

## Results

### Bilirubin content in plasma and cerebella

**Figure 1:**
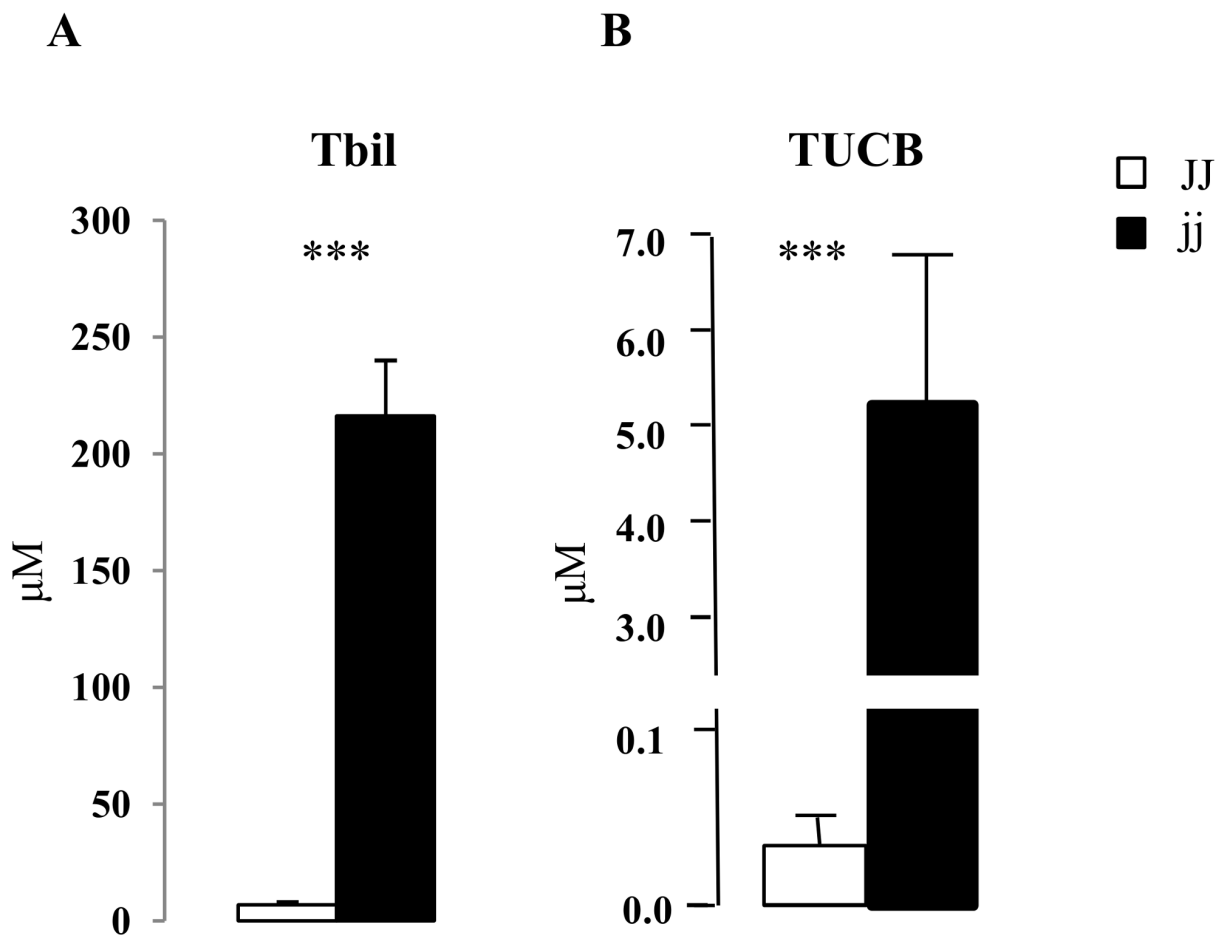
Plasma total unconjugated bilirubin amounts, and tissue cerebellar unconjugated bilirubin concentration. A) plasma total unconjugated bilirubin amounts (Tbil) , and B) tissue unconjugated bilirubin concentrations (TUCB). □ Normal homozygous JJ and ■ Hyperbilirubinemic homozygous jj Gunn rat. Data are expressed as mean ± SD. 6 animals of both genotypes were used. Statistical significance: *** p < 0.001.

Hyperbilirubinemia develops spontaneously in Gunn rats, due to the lack of efficient glucuronidation, resulting in marked unconjugated hyperbilirubinemia. Total plasma bilirubin was 216.25 ± 23.92 μM in jj pups (Tbil) as compared to 6.94 ± 1.28 μM in non-jaundiced JJ controls (p < 0.001) (Figure 1A). The unconjugated bilirubin (UCB) present in the circulation may enter tissues, diffusing across cellular membranes and the blood brain barrier, and accumulating in organs. In brain, this leads to neurological damage. In jj Gunn rats, the hyperbilirubinemia was associated with a 130 times higher bilirubin cerebellar concentration in jj pups (5200 ± 1600 *vs.* 40 ± 6 nM, jj *vs.* JJ rats, p < 0.001) (Figure 1B).

### mRNA expression of cell cycle regulators

**Figure 2:**
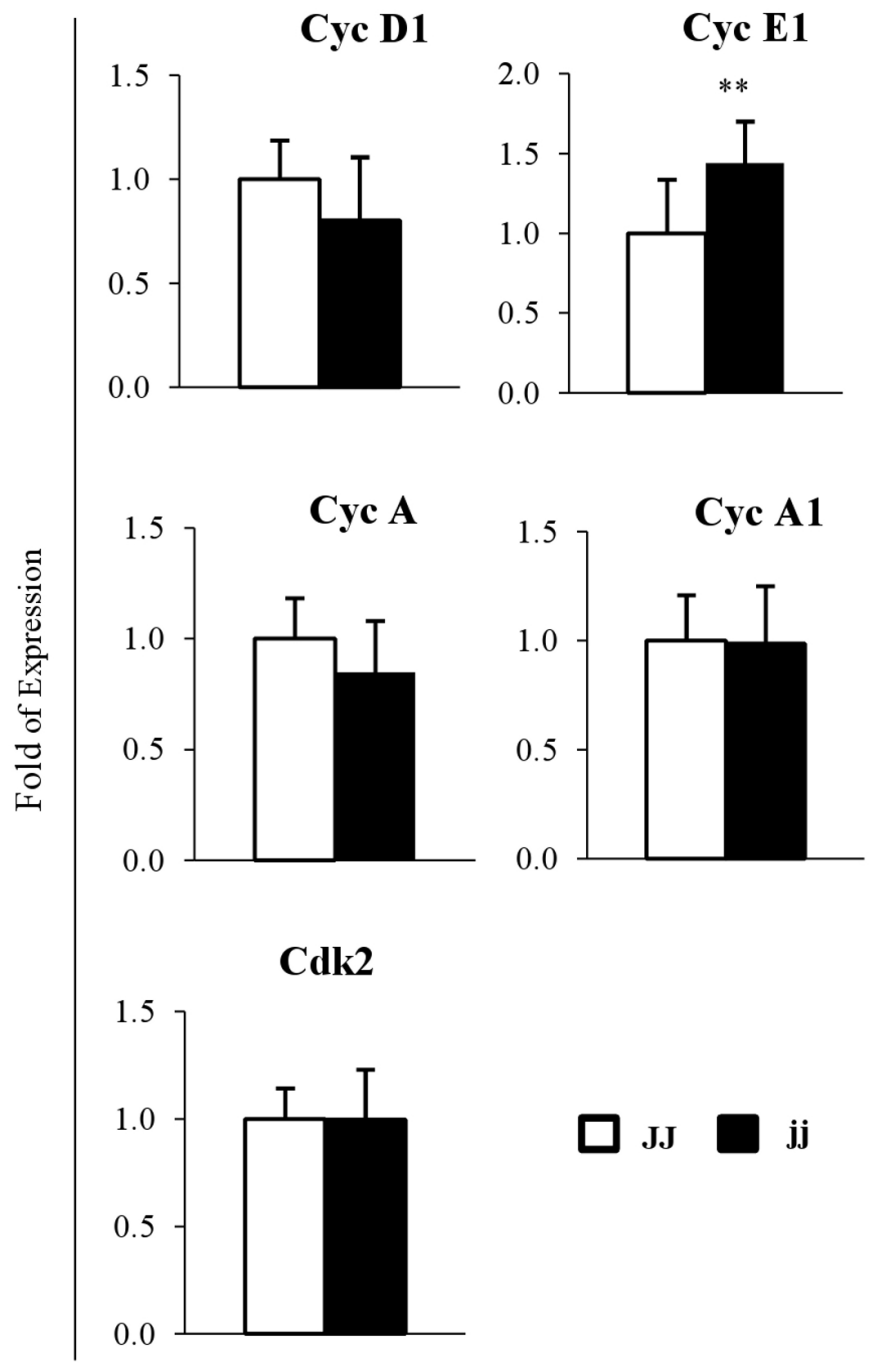
mRNA relative expression of Cyclin D1, Cyclin E1, Cyclin A, A1 and Cdk2. □ Normal homozygous JJ and ■ Hyperbilirubinemic homozygous jj Gunn rat. Data are expressed as mean ± SD. Statistical significance: ** p < 0.01.

Selected Cyclins and cyclin-dependent kinase 2 (Cdk2) expression was followed as markers of bilirubin influence on the cell cycle, as previously reported by others [31–34]. The expression of Cyclin D1 (the first cyclin to be induced, regulating the G_0_ to S phase transition) and Cyclin A (S phase transition) mRNA was reduced (20% and 15%, respectively) in cerebella of jj rats, whereas Cyclin A1 and Cdk2 (forming active complexes with the Cyclins here studied) mRNA levels were comparable to controls. By contrast, the Cyclin E1 (G1 to S phase) mRNA level was increased by 45% (p < 0.01) in hyperbilirubinemic jj pups (Figure 2).

### Protein expression of cell cycle regulators

**Figure 3:**
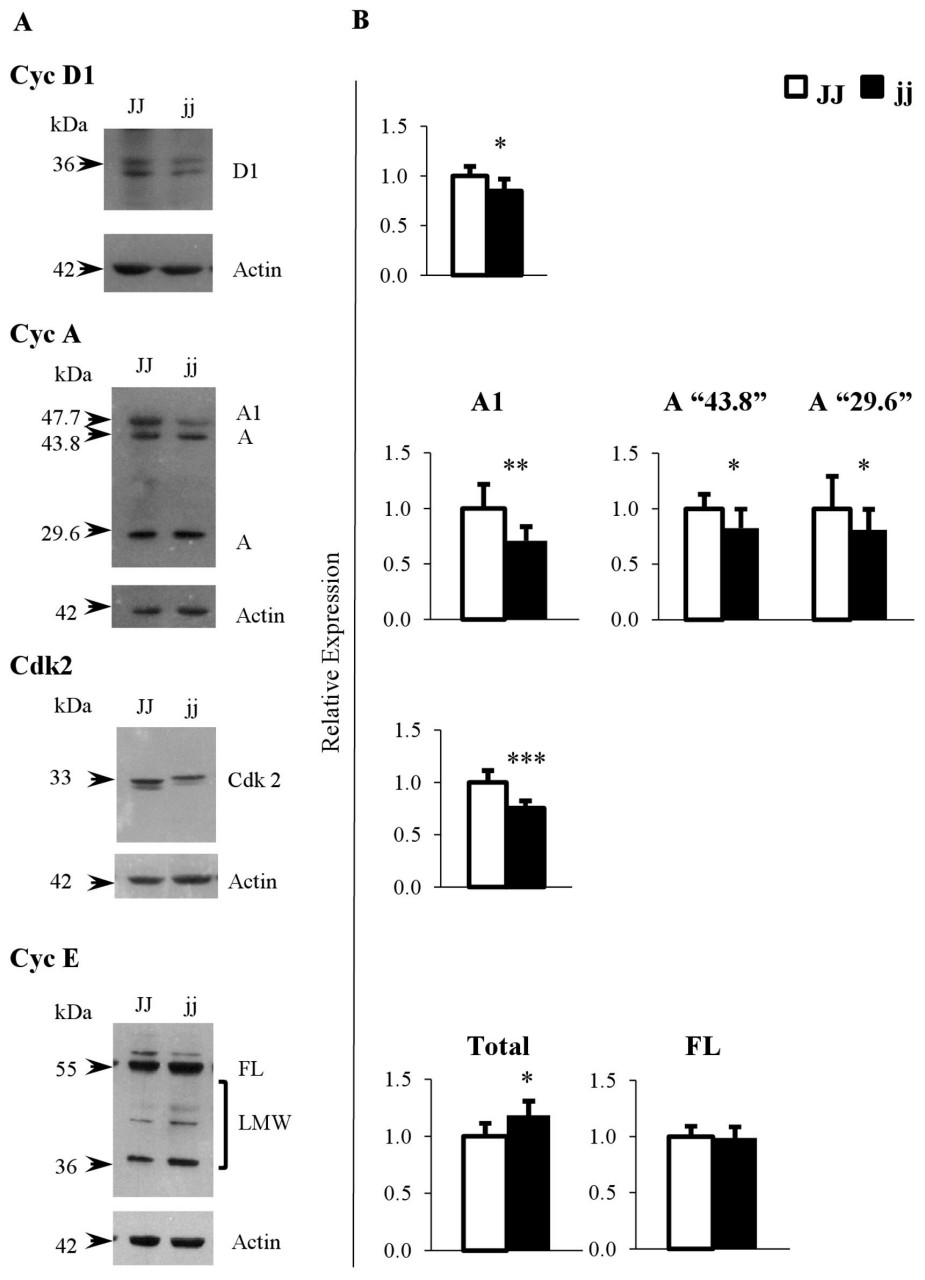
Representative Western blot and protein relative expression of Cyclin D1, Cyclin E, Cyclin A, A1 and Cdk2. A) Representative Western blot, and B) protein relative quantification. **FL**: full length Cyclin E (50 kDa), **LWM**: low molecular weight Cyclin E forms (49-34 kDa), **Total**: FL plus LMW Cyclin E forms. □ Normal homozygous JJ and ■ Hyperbilirubinemic homozygous jj Gunn rat. Data are expressed as mean ± SD. Statistical significance: * p < 0.05, ** p < 0.01, *** p < 0.001.

Similarly, the selected Cyclins and Cdk2 protein expression was evaluated. Figure 3A shows that the antibodies recognized well-defined band(s) at the expected molecular weight(s) in rat tissues (CycD1: 36 kDa; Cyclin A: 43.8 KDa and 29.6 KDa; Cyclin A1: 47.7 KDa, and Cdk2: 33 kDa). In addition to the expected Cyclin E band at 50 KDa (full length, FL), the E-4 antibody (N-terminal epitope) also detected the cleaved forms of Cyclin E ranging from 49 to 34 KDa (low molecular weight forms, LMW) [61,62].

Where the bands were quantified, a significant decrease in the level of Cyclin D1 (15%; p < 0.05), Cyclin A1 (about 30%; p < 0.01), and “43.8” and “29,6” Cyclin A rat isoforms (each about 20%; p < 0.05) were observed (Figure 3 B) in hyperbilirubinemic rats. Similarly, the level of Cdk2 was decreased by 25% (p < 0.001) in jj rats.

Total Cyclin E (FL plus LMW) detected by the E-4 antibody was increased about 19% (p < 0.05) in jj animals, due entirely to the increased amounts of the LMW cleaved forms of Cyclin E; while the amount of the FL form (50 kDa) was unchanged.

### p18-Cyclin E protein expression and cParp-1 and active Caspase3 analysis

**Figure 4:**
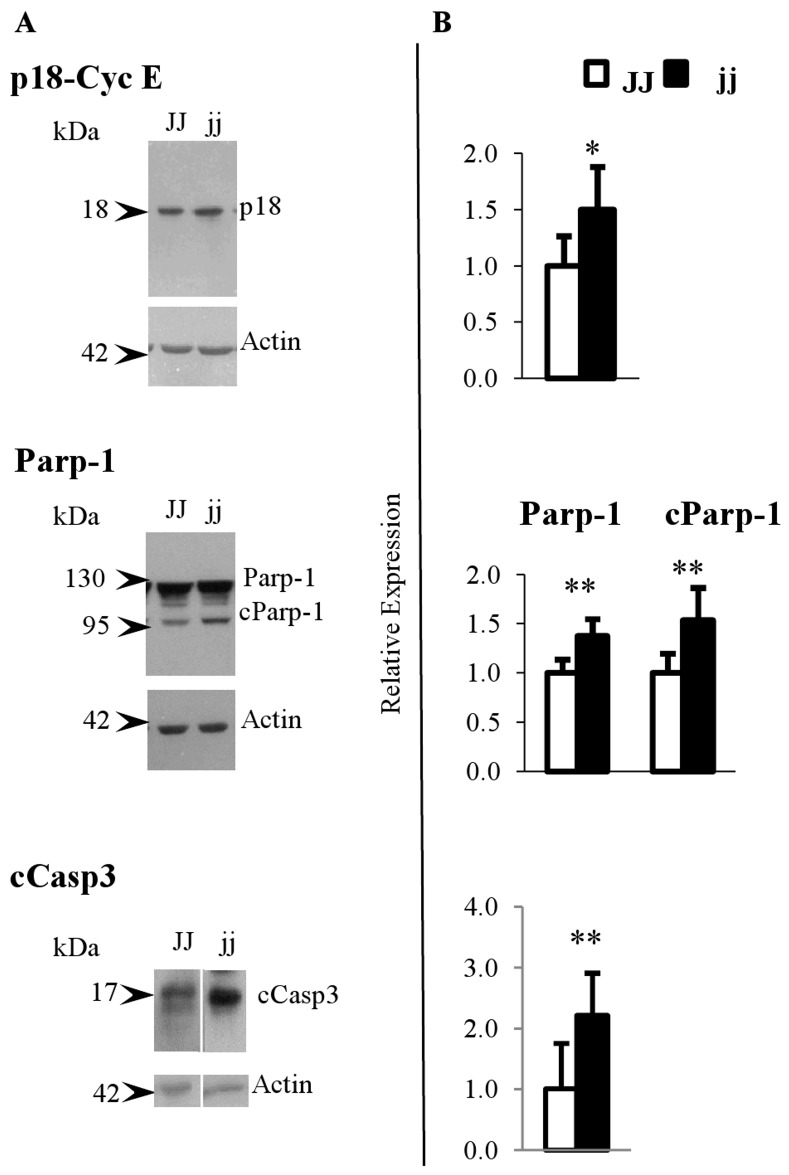
Representative Western blot and protein relative expression of p18-Cyclin E, Parp-1, cParp-1 and cCasp3. A) Representative Western blot, and B) protein relative quantification. **cParp-1**: cleaved Parp-1. **cCasp3**: active caspase3 □ Normal homozygous JJ and ■ Hyperbilirubinemic homozygous jj Gunn rat. Data are expressed as mean ± SD. Statistical significance: * p < 0.05, ** p < 0.01.

Because the increased expression of Cyclin E was unexpected and, apparently, opposite to the concept of cell cycle arrest, we decided to better analyse its up-regulation. For this reason, we used the M-20 antibody (C-terminal epitope) which recognize the p18-Cyclin E proteolytic fragment (Figure 4A), with known pro-apoptotic properties [63–65]. As shown in Figure 4B, the p18-Cyclin E level was increased by about 67% (p < 0.05). In agreement, Parp and cleaved Parp-1 (cParp-1, Figure 4A), a marker of early apoptosis [66,67], were significantly increased in jj animals by 38% and 54%, respectively (both p < 0.01) as compared to control (JJ) animals (Figure 4B). Under standard Western blot conditions, the protein expression of cCasp3 was only detectable in jj animals (data not shown). In order to perform the quantification, we obtained the signal by exposing the film longer. Under these conditions, we observed a cCasp3 amount double in hyperbilirubinemic pups *vs.* the JJ control (p < 0.01) (Figure 4B).

### Western blot analysis of cerebral cortex samples

Cerebral cortex samples were also studied as cortex represents a CNS post-mitotic tissue with tissue UCB amount similar to jj cerebellum [68]. Cdk2 was selected since significantly down regulated while Cyclin E, Parp-1; cParp-1 and cCasp3 were up regulated in the jj cerebellum. When the analysis was performed on cerebral cortex samples, the Cdk2, Cyclin E and Parp-1 levels were similar in the two genotypes (mean ± SD: Cdk2: JJ: 1.0 ± 0.07; jj: 0.96 ± 0.08; T.Test: 0.34; Cyclin E: JJ: 1.0 ± 0.3; jj: 0.89 ± 0.6; T.Test: 0.5; Parp-1: JJ: 1.0 ± 0.06; jj: 0.91 ± 0.12; T.Test: 0.17). Both cParp-1 / cCasp3 were not detectable neither in JJ or in jj Gunn rats cerebral cortex (data not shown). 

### FACS analysis of cell cycle

**Figure 5:**
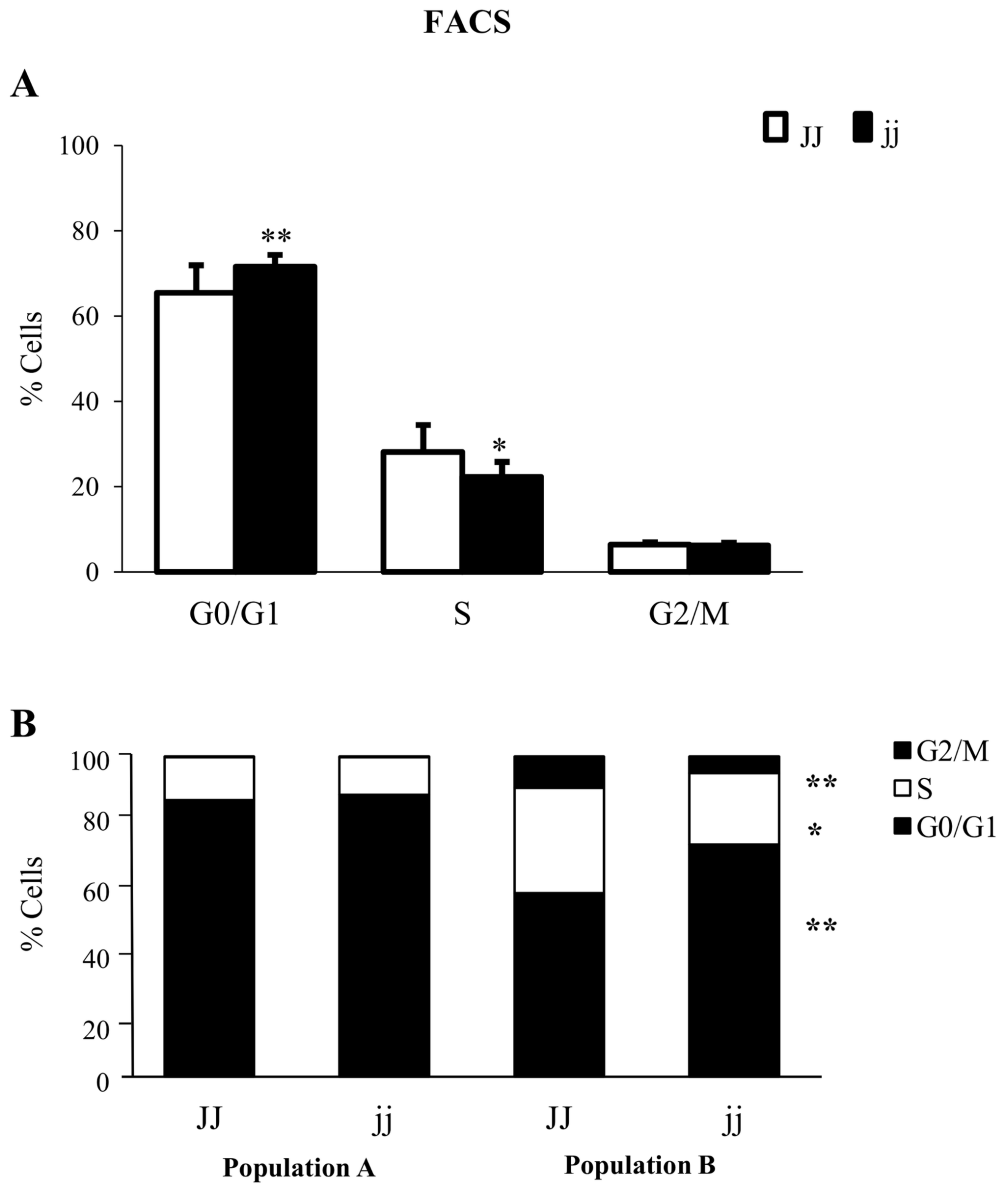
Cell Cycle profile in JJ and jj P9 Gunn rats. Cell cycle was analysed on cells from whole cerebella (**A**), and in population A and B, identified by forward- scattering (**B**). □ Normal homozygous JJ and ■ Hyperbilirubinemic homozygous jj Gunn rat. Data are expressed as mean ± SD. Statistical significance: * p < 0.05, ** p < 0.01.

To confirm the cell cycle perturbation *in vivo*, we performed a FACS analysis in the pool of dissociated cells from whole cerebella (Figure 5 A). In cerebella from hyperbilirubinemic jj animals as compared to cerebella from normobilirubinemic JJ rats, the proportion of cells in G0/G1 phase was increased (p < 0.01), while that in S phase was decreased (p < 0.05). On the contrary, the percentage of cells in the latest cell cycle phase (G2/M) was identical in both genotypes.

By forward scattering analysis, two population, namely population A and B, were detected (Figure 5 B). The cell cycle in population A was comparable in the two genotypes, while in population B, an increased presence of cells in G0/G1 phase (p < 0.01) was observed, with a concomitant decrease in S phase (p < 0.05), and G2/M phase (p < 0.01) (Figure 5 B). We did not identify the two population by specific antibodies; most probably because the dissociation procedure needed to obtain an optimal single cell suspension for FACS, disrupted the epitopes.

### *In vitro* analysis of bilirubin effects on the cell cycle and apoptosis

To identify which proliferating cerebellar population (neuronal or astroglial) was primarily affected by bilirubin, we used an *in vitro* model of primary cultures of granular and astrocyte cells obtained from Wistar rat cerebellum, and challenged the cells with toxic concentration (126 nM) of free bilirubin.

**Figure 6:**
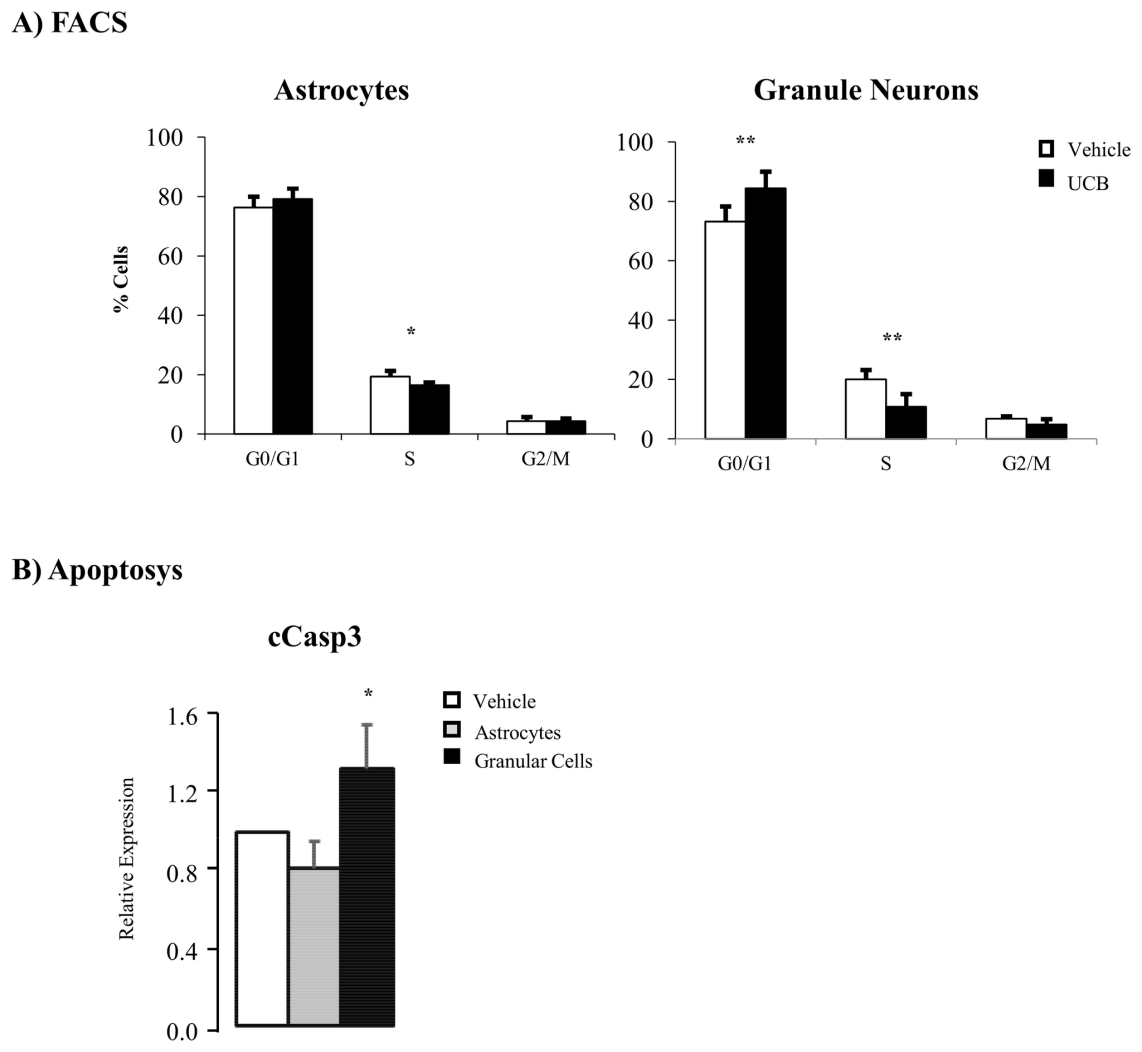
Effects of bilirubin on cell cycle and apoptosis in cerebellar granular and astrocytes primary cell cultures. **A**) Celll cycle analysis by FACS on cerebellar primary cell cultures exposed to 126 nM of Bf (black bars) and vehicle: 0.5% DMSO (white bars). **B**) Western blot analysis of cCasp3 expression in astrocytes (grey bars) and granular cells (black bars) primary cell cultures exposed to 126 nM of Bf *vs*. vehicle (0.5% DMSO, white bars). Data are expressed as mean ± SD. Statistical significance: * p < 0.05, ** p < 0.01.

By FCAS analysis, in granular cells we observed an increased percentage of cells in Go/G1 phase (p < 0.01), with a concomitant decrease in S and G2/M phases (p < 0.05 and p < 0.01, *vs.* vehicle alone, respectively). In astrocytes, a statistical difference (p < 0.05) was observed only in phase S, while no alterations of G0/G1 or G2/M phases were present (Figure 6 A). 

Similarly, apoptosis, as evaluated by Caspase3 levels, was increased only in granular cells exposed to bilirubin (p < 0.05), while no changes were detectable in astrocytes. 

## Discussion

Despite several mechanism(s) of bilirubin toxicity to CNS have been long proposed [20,22], the possibility that bilirubin may alter the cell cycle has emerged only more recently [31–35]. The proliferation of granular precursor cells in the external granular layer is the dominant event in the rodent cerebellar growth early after birth [38,39], rendering this structure vulnerable to bilirubin toxicity and suggesting a link between cell cycle perturbations and the typical cerebellar hypoplasia of the jj Gunn rat [13].

By the use of this animal model, we report at P9 an increased percentage of cells in late G0/G1 phase in the cerebellum of hyperbilirubinemic (jj) Gunn rat pups. The perturbation of the cell cycle was corroborated by the decreased *in vivo* expression of Cylins D1, A, and A1 protein, and of the constitutively expressed Cdk2. This was associated with a 130 fold higher UCB concentration in the cerebellum of jj pups, supporting the conclusion that high tissue concentration of UCB we observed *in vivo* interferes with the cell cycle of cerebellar cells, as previous reported *in vitro* for neurons [37] and other cells type [31–34]. The cerebellum of rodents rapidly develops early after birth, a period comparable to perinatal in humans [38]. Although cerebellar hypoplasia in humans is not usually reported, sporadically cases have been described [40], and signs of functional and structural findings of cerebellar bilirubin have been documented in humans [3,18,19]. This is most probably due to the fact the human cerebellum develops during the fetal life, and no mitotic cells are present after the birth when the bilirubin level picks. This hypostesis is supported by the lack of effects on both cell cycle and apoptosis in the Gunn cerebral cortex, a post-natal post mitotic CNS structure, having similar to cerebellum UCB content.

We observed a marked increase in Cyclin E mRNA and protein in jj animals. This was apparently in contrast with the concept that UCB arrests the cell cycle, and contrary to the down-regulation of Cyclin E reported *in vitro* by Ollinger [31], where primary cultures of vascular smooth muscle where treated with up to 200 μM bilirubin/biliverdin. This apparent disagreement may be justified by the relevant difference in experimental model used. In fact, the *in vivo* data were obtained on whole homogenates of P9 cerebella, an experimental model more close to in vivo situation of severe neonatal jaundice or Crigler-Najjar type I patients. The high levels of the total Cyclin E protein are almost entirely accounted by the increase in the LMW isoforms, whereas the protein expression of the FL form was unchanged. The latter result is in agreement with data reported by Peyton, where local administration of bilirubin to injured rat carotid arteries did not affect the Cyclin E FL protein amount [33]. Unfortunately, no information was provided on the levels of Cyclin E cleaved forms.

The complex of LMW Cyclin E with Cdk2 is known to be functionally more active then FL Cyclin E/Cdk2 complex, accelerating the cell cycle progression and proliferation [61,62], and replacing Cyclin D1 deficiency [69]. However, this seems not to be our case, as FACS analysis on cerebella of hyperbilirubinemic pups confirmed a significant increase in the G0/G1 cell populations with concomitant decrease of cells in S phase. It is important to consider that the samples we analyzed (Western blot, real time PCR, FACS) represent the entire pool of cells that constitute the cerebellum, and include cells more (neurones) and less (glial cells) sensitive to bilirubin toxicity [70–73]. The presence of the less sensitive cells such as population A in Figure 5 B, might partially mask the effect of bilirubin toxicity to most sensible cells (population B, Figure 5 B), and minimize the difference between the two genotypes. 

We reported a relevant increase (67%) for the p18-cyclin E fragment in jaundiced jj Gunn rats. This fragment is generated through caspase-mediated proteolytic cleavage of the C-terminal portion of Cyclin E, abrogating its binding to Cdk2 and consequent activation of Cyclin E activity [74]. Moreover, the p18-cyclin E fragment has a critical role in the amplification of the intrinsic apoptotic pathway by activating the caspase-mediated apoptotic cell death [63,64]. This involves cleavage of several proteins essential for cellular function and survival [75], one of which is Parp-1, Poly (ADP-ribose) polymerase-1, synthesized first and then cleaved (cParp, 85 kDa fragment) in the early steps of apoptosis [66,67,76]. In line with this is the increased level of cParp and cCasp3 in the cerebellum of P9 hyperbilirubinemic Gunn rat pups (Figure 4) indicating the enhancement of the apoptotic process by the higher UCB levels present in these jaundiced animals, as previously demonstrated in *in vitro* by the exposure of rat neurons to UCB [26]. Unfortunately, we were not able to identify the two population by specific antibodies or primers, most probably because the procedure we applied to obtain an optimal single cell suspension to be assessed by FACS, was disruptive, when we challenged primary neuronal and astrocytes cells with toxic amounts of bilirubin, we confirmed the higher sensitivity to bilirubin of neuronal granular cells as compared to astrocytes (Figure 6). Moreover, we demonstrated that bilirubin is the toxic compound for the perturbation of the cell cycle and apoptosis only in the granular cells, pointing as cell population target of bilirubin toxicity *in vivo*. 

In conclusion, our data obtained *in vivo* and confirmed *in vitro* indicate that the high level of UCB in the cerebellum of jaundiced jj Gunn rats causes cell cycle perturbation and the enhancement of apoptosis, that may account at least in part for the characteristic cerebellar hypoplasia of these animals. Cell cycle alteration and apoptosis appear to be intimately linked through an increased expression of p18-Cyclin E. This hypothesis is supported by the lack of effects in the cerebral cortex, a post mitotic CNS structure, in spite of a similar to cerebellum UCB content. Our data also show that cerebellar neuronal granular cells are the principal target of bilirubin neurotoxicity. Since these are the fist data collected *in vivo*, they may be important in understanding the mechanisms of bilirubin toxicity to the CNS in humans. 
